# Impact of Dietary Nutrients on the Prevalence of Dry Eye Syndrome among Korean Women Aged 40 and above: Evidence from the Korea National Health and Nutrition Examination Survey

**DOI:** 10.3390/nu16030372

**Published:** 2024-01-26

**Authors:** Jeong-Mee Kim, Yean-Jung Choi

**Affiliations:** 1 Department of Visual Optics, Far East University, Eumseong 27601, Republic of Korea; jmkim@kdu.ac.kr; 2 Department of Food and Nutrition, Sahmyook University, 815, Hwarang-ro, Nowon-gu, Seoul 01795, Republic of Korea

**Keywords:** dry eye syndrome, prevalence, Korean women, dietary nutrients, Korea national health and nutrition examination survey (KNHANES), nutrient-rich diet, prevention strategy

## Abstract

This study aimed to assess the prevalence of dry eye syndrome among Korean women aged 40 and above and explore the correlation between the syndrome and daily dietary nutrient intake. We analyzed data from 92,888 female participants (mean age: 63.35 ± 8.86 years) from the 8th Korea National Health and Nutrition Examination Survey (KNHANES 2019). Dietary intake was evaluated using a personalized 24 h recall method for 21 nutrients, including macronutrients, macro- and micro-minerals, and both water- and fat-soluble vitamins. Associations between nutrient intake and dry eye syndrome were determined using odds ratios from multivariate logistic regression. We found a 7.7% prevalence of dry eye syndrome in the population studied. Intake of dietary fiber (adjusted OR: 0.72), protein (adjusted OR: 0.84), omega-3 fatty acids (adjusted OR: 0.90), water (adjusted OR: 0.76), calcium (adjusted OR: 0.82), phosphate (adjusted OR: 0.87), potassium (adjusted OR: 0.88), magnesium (adjusted OR: 0.87), vitamin A (adjusted OR: 0.78), vitamin C (adjusted OR: 0.73), and vitamin E (adjusted OR: 0.86) was inversely associated with dry eye syndrome prevalence (*p* < 0.0001 for all). Conversely, a higher intake of carbohydrates (adjusted OR: 1.23), sugar (adjusted OR: 1.30), fat (adjusted OR: 1.25), cholesterol (adjusted OR: 1.32), sodium (adjusted OR: 1.18), iron (adjusted OR: 1.28), and zinc (adjusted OR: 1.26) correlated with an increased risk (*p* < 0.0001 for all). No significant associations were found between the prevalence of dry eye syndrome and the intake of omega-6 fatty acids and vitamin D. Our study identified significant associations between specific dietary nutrients and the risk of dry eye syndrome among Korean women aged 40 and above. These findings suggest that dietary choices could influence the likelihood of developing dry eye syndrome, indicating a potential role for dietary intervention in its management. However, it is important to note that these observations are preliminary, and further research is necessary to confirm these relationships and explore their implications for dietary recommendations in dry eye syndrome prevention and management.

## 1. Introduction

Dry Eye Syndrome (DES), or keratoconjunctivitis sicca, presents a multifaceted challenge to ocular health, marked by a disrupted tear film and damage to the ocular surface [1,2,3]. This complex condition results from a confluence of factors such as tear film instability, inflammation, epithelial injury, and various external contributors, including age, gender, hormonal fluctuations, environmental conditions, lifestyle choices, and systemic ailments like autoimmune diseases [4].

In South Korea, where the population is aging rapidly, there has been an increasing focus on health concerns, especially among women over 40 who are highly economically active. DES, characterized by intense discomfort, sensitivity to light, and diminished vision, has risen to prominence as a critical public health issue [1,5]. According to the Health Insurance Review and Assessment Service, the past decade has seen a 31.7% increase in DES patients in Korea, with consistent annual growth. Particularly noteworthy is the high incidence among Koreans in their 40s and 50s, making up 34.7% of these cases, and a notably higher prevalence among women compared to men [6]. The impact of DES is more pronounced in women than men both in terms of prevalence and severity. Left unmanaged, it can significantly impair quality of life [4,7,8,9,10,11].

Emerging research underscores the importance of specific nutrients, including omega-3 fatty acids, vitamins A and D, and antioxidants, in maintaining ocular surface health. This insight has spurred interest in the role of dietary nutrients in alleviating and potentially treating DES [12,13,14,15,16], as evidenced by the increasing adoption of nutritional supplements for eye health [15,16].

While the connection between diet and DES is gaining attention, the specific relationship between daily nutrient intake and this condition requires more exploration. A deeper understanding of dietary patterns and nutritional intake within this demographic could be vital in preventing and managing DES. Therefore, this study aims to investigate the prevalence of DES among Korean women over 40 and examine the link between their daily dietary nutrients and this syndrome.

## 2. Materials and Methods

### 2.1. Data Resource and Study Population

The Korea National Health and Nutrition Examination Survey (KNHANES) employs a detailed and systematic approach to assess the health and nutritional status of the Korean population. Annually, 25 households from 192 regions are selected as a probability sample, surveying approximately 10,000 individuals aged 1 year and above. Participants are categorized into children (1–11 years old), adolescents (12–18 years old), and adults (19 years and older), with survey items tailored to each group’s life cycle stage. Surveys are conducted year-round in four regions weekly, with a specialized team operating a mobile examination vehicle for comprehensive health checkups and surveys. The check-up involves measurements concerning chronic diseases (obesity, hypertension, diabetes, and dyslipidemia) using height, weight, blood pressure, and blood and urine tests, along with oral examinations. The health survey assesses behaviors like smoking, drinking, physical activity, and mental health via questionnaires. Approximately a week later, a nutritional survey is conducted at the participant’s home, focusing on dietary habits and food consumption.

**Figure 1:**
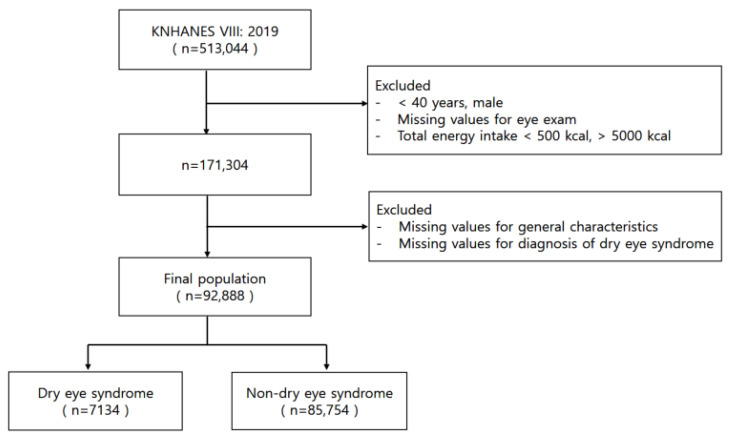
Selection process flowchart for study participants.

This study analyzed data from the 8th Korea National Health and Nutrition Examination Survey (KNHANES VIII) conducted in 2019 by the Korea Centers for Disease Control and Prevention, focusing on women aged 40 and above from its pool of 513,044 participants. We excluded those with incomplete eye examination data, extreme daily energy intakes (below 500 kcal or above 5000 kcal), or missing information on key variables or dry eye syndrome diagnosis. The final sample included 92,888 women, who were divided into groups based on the presence or absence of dry eye syndrome (Figure 1).

### 2.2. Socio-Demographic and Health Variables

We collected socio-demographic data covering age, BMI (classified as underweight, normal, overweight, or obese), education (below high school or high school and above), household income (low, middle, or high), and occupation (white-collar, worker, indoor, and outdoor). Near-work activity duration (<1 h, 1–3 h, and ≥4 h) and menopausal status were also documented. The presence of systemic diseases like rheumatoid arthritis, osteoporosis, diabetes, and thyroid disease was determined based on medical diagnoses.

### 2.3. Diagnosis of Dry Eye Syndrome and Dietary Nutrient Intake Assessment

In this study, the identification of dry eye syndrome (DES) among the participants was conducted through structured interview-based surveys, adhering to diagnostic criteria established by ophthalmologists. We stratified female participants over the age of 40 into four distinct age groups—40–49, 50–59, 60–69, and 70+ years of age—to analyze variations in DES prevalence.

For dietary data collection, KNHANES uses direct interviews to delve into the participants’ eating patterns, food consumption frequency, and specific nutrient intake based on a 24 h recall method. This dietary survey is characterized by its open-ended format, allowing individuals to freely list all foods and beverages consumed on the previous day. The 24 h recall process is meticulous, beginning with identifying meals and food items, followed by quantifying consumption using measuring tools like cups and spoons. Detailed inquiries into food preparation methods and seasonings are made, ensuring a thorough understanding of dietary habits. This is supplemented by a verification step carried out to confirm the accuracy of the food information collected, and if necessary, an additional survey is conducted for further clarification. This methodical approach ensures a precise capture of each participant’s dietary intake over a single day.

The daily nutrient intake is then calculated by aggregating the intake from all food and nutrient sources reported during the recall period. To assess the adequacy of the nutrient intake, we refer to the Korean Dietary Reference Intakes (KDRI) 2020 guidelines, which provide benchmarks for sufficient or excessive nutrient consumption based on age and gender. Our assessment focuses on a comprehensive range of 21 nutrients, encompassing macronutrients, essential major and trace minerals, and water-soluble and fat-soluble vitamins, derived from the detailed 24 h recall data.

### 2.4. Statistical Analysis

The data were processed using SAS software version 9.4. Our analysis adhered to the complex sample design methodology, incorporating clustering, stratification, and weighting, mirroring the approach employed by the 7th Korea National Health and Nutrition Examination Survey. Descriptive statistics were presented as means ± standard deviation for continuous variables and frequencies for categorical variables. The chi-square test was used for categorical comparisons between groups with and without DES, while ANCOVA was applied to continuous variables. Logistic regression (both univariate and multivariate) was used to explore the association between dry eye risk factors and dietary nutrients, with findings presented as odds ratios and 95% confidence intervals. A *p*-value below 0.05 was deemed significant.

## 3. Results

### 3.1. Study Population and Characteristics

Our study comprised 92,888 women aged 40 and above, with a mean age of 63.35 ± 8.86 years. The overall prevalence of dry eye syndrome (DES) was 7.7%. The highest prevalence of DES was observed among women in their 50s, constituting 63.1% of the DES group. The socio-demographic factors and systemic diseases for both the DES and non-DES groups are detailed in Table 1. A higher prevalence of DES was associated with greater levels of education, household income, and the proportion of white-collar workers, especially those with longer durations of near work. The incidence rates of menopause and diabetes were comparable between the groups. However, a higher incidence of rheumatoid arthritis and thyroid disease was observed in the DES group, while osteoporosis was more prevalent in the non-DES group.

### 3.2. Risk Factors for Dry Eye Syndrome

In adjusted Model 2, incorporating age, BMI, residential area, educational level, household income, occupational category, near work, current smoking habit, and heavy alcohol drinking, several factors were associated with an increased likelihood of DES. These included being in the 50s age bracket (OR: 1.81), being underweight (Adjusted OR: 1.74), having an education beyond high school (Adjusted OR: 1.52), and engaging in 1–3 h of near work (Adjusted OR: 1.46). Conversely, a higher household income (Adjusted OR: 0.67) and being an outdoor worker (Adjusted OR: 0.61) were associated with a reduced prevalence of DES (Table 2).

In adjusted Model 2, which incorporated age, menopause, medications, rheumatoid arthritis, osteoporosis, diabetes, thyroid disease, and refractive surgery, menopause (Adjusted OR: 1.31), rheumatoid arthritis (Adjusted OR: 1.54), osteoporosis (Adjusted OR: 1.47), diabetes (Adjusted OR: 1.41), and thyroid disease (Adjusted OR: 1.41) were all identified as factors contributing to a heightened risk of DES (Table 3).

### 3.3. Association between Daily Intake of Dietary Nutrients and Dry Eye Syndrome

Supplementary Table S1 delineates the differences in average daily nutrient intake between individuals with and without DES. It shows that those with DES had a slightly higher intake of carbohydrates, averaging 263.45 ± 97.42 g daily, compared to 259.71 ± 99.77 g in the non-DES group. Protein intake was also higher in the DES group, with an average increase of 6.28 g per day. Additionally, this group consumed more various types of fats, including saturated, monounsaturated, and polyunsaturated fat; N6 fatty acids; and cholesterol. In terms of mineral intake, the DES group had a higher daily consumption of essential minerals. Specifically, they consumed an average of 0.71 mg more iron and 13.24 mg more magnesium than those without DES. Regarding vitamins and sugars, there was an increased consumption of sugar, vitamin A (20.92 μg RAE higher per day), carotene, vitamins B1 and B2, niacin, folate, and vitamin E in the DES group. Conversely, the daily intake of N3 fatty acids was 0.15 g less in the DES group, and this group also presented a decrease in calcium intake by 34.7 mg per day. The consumption of retinol and vitamin D was also lower in this group. Notably, there were no significant differences in the intake of fiber and vitamin C between the two groups.

Our investigation into the relationship between dietary nutrient intake and DES considered both unadjusted (Model 1) and adjusted factors (Model 2), which included age, BMI, educational level, household income, occupational category, near work, menopause, and systemic diseases. Our analysis indicated that certain nutrients influenced the likelihood of developing DES (Table 4). Among the 21 nutrients analyzed, a higher intake of fiber (Adjusted OR: 0.72), protein (Adjusted OR: 0.84), omega-3 fatty acids (Adjusted OR: 0.90), water (Adjusted OR: 0.76), calcium (Adjusted OR: 0.82), phosphate (Adjusted OR: 0.87), potassium (Adjusted OR: 0.88), magnesium (Adjusted OR: 0.87), vitamin A (Adjusted OR: 0.78), carotene (Adjusted OR: 0.82), vitamin C (Adjusted OR: 0.73), and vitamin E (Adjusted OR: 0.86) was found to be protective against DES (all *p* < 0.0001). On the other hand, greater consumption of carbohydrates (Adjusted OR: 1.23), sugar (Adjusted OR: 1.30), fat (Adjusted OR: 1.25), cholesterol (Adjusted OR: 1.32), sodium (Adjusted OR: 1.18), iron (Adjusted OR: 1.28), and zinc (Adjusted OR: 1.26) was associated with an increased prevalence of DES (all *p* < 0.0001). This study did not find a significant association between the intake of omega-6 fatty acids, vitamin D, and the prevalence of DES.

## 4. Discussion

In our study, we investigated the prevalence of dry eye syndrome (DES) among Korean women over 40 and its potential link to daily dietary nutrients. This research contributes new insights to the field by examining a demographic that has been less represented in previous studies. We found that age-related factors and medical conditions like menopause, rheumatoid arthritis, osteoporosis, diabetes, and thyroid disease are significant risk factors for DES. DES’s increased risk with age is attributed to physiological changes impacting tear composition and production [17]. Particularly in women over 40, the decline in estrogen—which plays a crucial role in ocular moisture and lubrication—after menopause can lead to DES [4,18,19]. Moreover, the use of specific medications, including antihistamines, decongestants, and antidepressants, is known to exacerbate DES symptoms [20]. The higher prevalence of autoimmune conditions like rheumatoid arthritis and Sjögren’s syndrome in women also contributes to the increased incidence of severely dry eyes [21,22]. External factors such as long-term contact lens wear, corneal refractive surgery, and environmental conditions in workplaces—characterized by controlled climates and extended screen time—further influence the stability of the tear film and exacerbate DES symptoms [23,24,25,26]. The effective treatment of DES, therefore, requires a comprehensive approach that addresses the unique combination of systemic, hormonal, environmental, and lifestyle factors contributing to each individual’s condition.

The adage “You are what you eat” underscores the importance of diet not only in energy provision but also in disease management, which is particularly relevant for ocular health [27]. Our findings reveal that certain nutrients, including fiber, protein, omega-3 fatty acids, water, and vitamins A, C, and E, are associated with a lowered risk of DES. Omega-3 fatty acids are notable for their anti-inflammatory properties, which are beneficial for corneal health [28,29]. Studies have indicated that women with DES have lower blood levels of omega-3 fatty acids compared to those without this condition [30], supporting the notion that increased intake of these fatty acids correlates with a reduced occurrence of DES among women [13,30,31,32,33]. Although dietary sources contribute to omega-3 levels, an optimal intake is often achieved through oral supplements. Similarly, vitamin A is crucial for the ocular surface epithelium, and deficiencies are linked to DES [34,35]. In young adult men with DES, short-term oral vitamin A supplementation improved tear quality, although quantity was not improved [15]. Vitamins E and C, both potent antioxidants, play a protective role within the eye. Vitamin E safeguards against the oxidation of fatty acids, while vitamin C assists in regenerating other antioxidants [36], including vitamin E, and supports vascular and connective tissue integrity within the eye, guarding against damage from free radicals [36]. Furthermore, vitamin E has been shown to boost the antioxidant capacity of lutein, providing protection for the retina [37]. The antioxidant effects of vitamins C and E may extend to enhancing ocular surface health, as evidenced by improved tear stability, volume, and subjective clinical symptoms [16]. Thus, our findings advocate for a diet rich in specific nutrients and, where appropriate, supplemented with oral antioxidants to mitigate the risk and symptoms of DES.

The tear film is a complex structure comprising three distinct layers: the outermost lipid layer, produced by the meibomian glands; the middle aqueous layer, secreted by the lacrimal glands; and the innermost mucus layer, produced by goblet cells in the conjunctiva [38]. These layers collectively protect the ocular surface and maintain corneal hydration. Nutrients like omega-3 fatty acids and vitamins A, C, and E contribute to tear film stability and protect against inflammation and oxidative stress. For instance, omega-3 fatty acids, when integrated into the lipid layer, bolster its integrity, thus mitigating premature tear evaporation [36]. Vitamin A plays a pivotal role in mucin production, which imparts a gel-like consistency to the tear film, essential for its stabilizing function [38]. Meanwhile, vitamins C and E, as antioxidants, defend the tear film against oxidative damage from free radicals [36]. Hence, the anti-inflammatory and antioxidative properties of these nutrients could ameliorate DES symptoms by dampening inflammation and shielding ocular tissues from oxidative harm [39]. Conversely, we observed that high consumption of carbohydrates, sugar, fat, cholesterol, iron, and zinc correlates with an increased prevalence of DES. Specifically, high levels of cholesterol may lead to dysfunction in the Meibomian glands, altering the lipid layer of the tear film [40,41]. A diet high in fat could impede the lacrimal gland’s secretory function [42]. Moreover, excessive sodium levels may induce dehydration, contributing to the manifestation of DES.

Regarding omega-6 fatty acids and vitamin D, our study did not demonstrate a significant association with DES prevalence. Prior research suggested that omega-6 fatty acids might alleviate DES, yet we did not observe a direct link [13,43,44,45]. While omega-6 fatty acids are crucial for health, an imbalance with omega-3 fatty acids intake may promote inflammation [13]. Hence, it is essential to maintain a proper balance of these fatty acids for ocular health. As for vitamin D, despite its role in immune modulation and calcium homeostasis and its potential anti-inflammatory effects on ocular surface diseases like DES [46,47], our results did not support a direct relationship between dietary vitamin D and DES prevalence. This finding contrasts with a meta-analysis by Liu et al. in which it was indicated that vitamin D deficiency correlates with aggravated subjective DED symptoms and diminished tear production [48]. Furthermore, other studies have reported that patients with DES had significantly lower serum vitamin D levels and that supplementation could ameliorate DES by elevating these levels [14,49]. Although dietary sources are less common, clinical and experimental evidence frequently acknowledges the benefits of oral vitamin D supplementation with respect to reducing DES prevalence [14,46,50].

Maintaining a well-rounded diet replete with diverse fruits, vegetables, whole grains, lean proteins, and healthy fats is paramount for overall eye health. Korean dietary practices are well positioned to provide an ample intake of nutrients that may mitigate the risk of dry eye syndrome. These nutrients include fiber, protein, omega-3 fatty acids, and vitamins such as vitamin A, C, and E [31]. Foods rich in these nutrients, such as blue-backed fish, perilla seeds, seaweed, eggs, sweet potatoes, and green and yellow vegetables, as well as strawberries, nuts, and beans, are commonly found in the Korean diet. It is advisable to regularly integrate these foods into one’s diet for their potential eye health benefits.

This study, while comprehensive, has certain limitations. It relies on self-reported dietary data, which can be prone to recall bias. Additionally, the cross-sectional design of this study limits our ability to draw definitive conclusions about causality. To understand the impact of diet on dry eye syndrome more conclusively and enhance dietary guidelines, future research, including randomized controlled trials and longitudinal studies, is essential. The exact mechanisms by which diet affects dry eye syndrome are yet to be fully understood, but it is believed that specific nutrients may influence tear production, composition, and stability, as well as protect the ocular surface from inflammation and oxidative damage. Further, in our endeavor to include a broad spectrum of participants with different types of dry eye syndrome, we did not limit our study to specific etiologies or severities. However, the involvement of an ophthalmologist to diagnose and categorize dry eye syndrome into clinically relevant categories would address some issues related to self-reporting. Nonetheless, even this method might not completely capture the clinical complexity of this condition, underscoring the need for more nuanced diagnostic approaches in future studies.

Our study uniquely linked specific nutrients, such as fiber, protein, omega-3 fatty acids, and vitamins A, C, and E, to a lower risk of DES in this demographic, highlighting the importance of diet in ocular health and DES management. We discovered that not only age-related factors and medical conditions but also dietary patterns contribute to DES. Particularly, our findings shed light on the positive impact of certain nutrients on eye health, which is a novel contribution in the context of older women and hormonal changes. Conversely, our research also indicates the negative effects of the high consumption of certain elements like carbohydrates, fat, and cholesterol on DES, providing new insights into dietary influences on eye health. Despite the limitations of our study, such as its use of self-reported data and its cross-sectional nature, our findings are crucial for understanding the diet–DES relationship. They pave the way for future research to develop more targeted dietary guidelines for DES prevention and management.

## 5. Conclusions

Our research focused on the multifaceted factors influencing the prevalence of dry eye syndrome among Korean women aged 40 and above. We discovered that certain dietary nutrients are linked to either a decreased or increased risk of this condition. These findings underscore the importance of considering a comprehensive range of factors in managing and preventing dry eye syndrome, including age, lifestyle, environmental influences, systemic diseases, and, notably, dietary choices. This study highlights the potential of dietary modification as a strategy for dry eye syndrome management. However, further research is imperative to establish specific dietary guidelines and determine the optimal nutrient intake for the effective prevention and treatment of dry eye syndrome. This future research will be crucial in developing comprehensive dietary recommendations to aid in the management and prevention of dry eye syndrome.

## Figures and Tables

**Table 1:** Characteristics of the participants categorized by dry eye syndrome from KNHANES 2019.

	Total	DES *	Non-DES
Number	92,888	7134 (7.7%)	85,754 (92.3%)
Age (years)	63.35 ± 8.86 **	58.52 ± 7.26	63.76 ± 8.87
Age (years)			
40–49	3608 (3.9%)	356 (5.0%)	3252 (3.8%)
50–59	31,674 (34.1%)	4499 (63.1%)	27,175 (31.7%)
60–69	33,254 (35.8%)	1580 (22.2%)	31,674 (36.9%)
70+	24,352 (26.2%)	699 (9.8%)	23,653 (27.6%)
BMI *** (kg/m^2^)			
Underweight	2297 (2.5%)	210 (2.9%)	2087 (2.4%)
Normal	59,535 (64.1%)	4866 (68.2%)	54,669 (63.8%)
Overweight	27,219 (29.3%)	1941 (27.2%)	25,278 (29.5%)
Obese	3837 (4.1%)	117 (1.7%)	3720 (4.3%)
Educational level			
Less than high school	47,068 (50.7%)	2167 (30.4%)	44,901 (52.4%)
More than high school	45,820 (49.3%)	4967 (69.6%)	40,853 (47.6%)
Household income			
Lower	39,633 (42.7%)	2569 (36.0%)	37,064 (43.2%)
Middle	17,798 (19.1%)	1313 (18.4%)	16,485 (19.2%)
Higher	35,457 (38.2%)	3252 (45.6%)	32,205 (37.6%)
Occupational category			
White-collar worker	9204 (9.9%)	1458 (20.5%)	7746 (9.0%)
Indoor worker	18,661 (20.1%)	1728 (24.2%)	16,933 (19.8%)
Outdoor worker	65,023 (70.0%)	3948 (55.3%)	61,075 (71.2%)
Near work			
≤1 h/D	52,957 (57.0%)	2760 (38.7%)	50,197 (58.5%)
1–3 h/D	27,862 (30.0%)	3120 (43.7%)	24,742 (28.9%)
≥4 h/D	12,069 (13.0%)	1254 (17.6%)	10,815 (12.6%)
Menopause			
Yes	84,965 (91.5%)	6485 (90.9%)	78,480 (91.5%)
No	7923 (8.5%)	649 (9.1%)	7274 (8.5%)
Rheumatoid arthritis			
Yes	3557 (3.8%)	378 (5.3%)	3179 (3.7%)
No	89,331 (96.2%)	6756 (94.7%)	82,575 (96.3%)
Osteoporosis			
Yes	20,546 (22.1%)	1382 (19.4%)	19,164 (22.4%)
No	72,342 (77.9%)	5752 (80.6%)	66,590 (77.6%)
Thyroid disease			
Yes	7937 (8.5%)	900 (12.6%)	7037 (8.2%)
No	84,951 (91.5%)	6234 (87.4%)	78,717 (91.8%)
Diabetes			
Yes	12,311 (13.3%)	947 (13.3%)	11,364 (13.3%)
No	80,577 (86.7%)	6187 (86.7%)	74,390 (86.7%)

* DES; Dry eye syndrome, ** mean ± standard deviation (SD), and *** BMI; body mass index.

**Table 2:** Logistic regression analysis of risk factors for developing dry eye syndrome among Korean women.

	Model 1 ^(1)^		Model 2 ^(2)^	
	OR [95% CI]	*p*-Value	OR [95% CI]	*p*-Value
Age (years)				
40–49	1		1	
50–59	1.51 [1.35–1.70]	<0.0001	1.81 [1.58–2.07]	<0.0001
60–69	0.46 [0.40–0.51]	<0.0001	0.77 [0.64–0.94]	<0.0001
70+	0.27 [0.24–0.31]	<0.0001	0.60 [0.45–0.80]	<0.0001
BMI (kg/m^2^)				
Underweight	1.13 [0.98–1.31]	<0.0001	1.74 [1.47–2.05]	<0.0001
Normal	1		1	
Overweight	0.86 [0.82–0.91]	0.0006	0.74 [0.68–0.81]	0.9664
Obese	0.35 [0.29–0.43]	<0.0001	0.23 [0.18–0.30]	<0.0001
Educational level				
Less than high school	1		1	
Beyond high school	2.52 [2.39–2.65]	<0.0001	1.52 [1.42–1.62]	<0.0001
Household income				
Lower	1		1	
Middle	1.15 [1.07–1.23]	0.1215	0.74 [0.69–0.80]	0.0025
Higher	1.46 [1.38–1.54]	<0.0001	0.67 [0.62–0.71]	<0.0001
Occupational category				
White-collar worker	1		1	
Indoor worker	0.54 [0.50–0.58]	0.0097	0.66 [0.61–0.72]	<0.0001
Outdoor worker	0.34 [0.32–0.37]	<0.0001	0.61 [0.57–0.66]	<0.0001
Near work				
≤1 h/D	1		1	
1–3 h/D	2.29 [2.17–2.42]	<0.0001	1.46 [1.37–1.55]	<0.0001
≥4 h/D	2.11 [1.97–2.26]	<0.0001	1.00 [0.92–1.08]	<0.0001

^(1)^ Model 1: Non-Adjusted (Crude). ^(2)^ Model 2: Adjusted for age, gender, BMI, residential area, educational level, household income, occupational category, near work, current smoking, and heavy alcohol drinking. OR; odds ratio, CI; confidence interval.

**Table 3:** Logistic regression analysis of risk factors for developing dry eye syndrome among Korean women according to systemic diseases and other variables.

	Model 1 ^(1)^		Model 2 ^(2)^	
	OR [95% CI]	*p*-Value	OR [95% CI]	*p*-Value
Menopause				
Yes	0.93 [0.85–1.01]	0.0741	1.31 [1.20–1.43]	<0.0001
No	1		1	
Rheumatoid arthritis				
Yes	1.45 [1.30–1.62]	<0.0001	1.54 [1.37–1.72]	<0.0001
No	1		1	
Osteoporosis				
Yes	0.84 [0.79–0.89]	<0.0001	1.47 [1.37–1.57]	<0.0001
No	1		1	
Diabetes				
Yes	1.00 [0.93–1.08]	0.9568	1.41 [1.29–1.53]	<0.0001
No	1		1	
Thyroid disease				
Yes	1.62 [1.50–1.74]	<0.0001	1.41 [1.31–1.53]	<0.0001
No	1		1	

^(1)^ Model 1: Non-Adjusted (Crude). ^(2)^ Model 2: Adjusted for age, menopause, medications, rheumatoid arthritis, osteoporosis, diabetes, thyroid disease, and refractive surgery. OR; odds ratio, CI; confidence interval.

**Table 4:** Multivariable logistic regression analysis of odds ratios for dietary nutrient intake and dry eye syndrome.

Nutrients	Model 1 ^(1)^		Model 2 ^(2)^	
	OR [95% CI]	*p*-Value	OR [95% CI]	*p*-Value
Carbohydrate (g)	1.31 [1.25–1.38]	<0.0001	1.23 [1.17–1.29]	<0.0001
Sugar (g)	1.59 [1.51–1.67]	<0.0001	1.30 [1.24–1.37]	<0.0001
Fiber (g)	0.88 [0.84–0.92]	<0.0001	0.72 [0.69–0.76]	<0.0001
Protein (g)	1.10 [1.05–1.16]	0.0001	0.84 [0.80–0.88]	<0.0001
Fat (g)	1.63 [1.55–1.71]	<0.0001	1.25 [1.18–1.32]	<0.0001
ω-3 fatty acid (g)	1.09 [1.04–1.14]	0.0006	0.90 [0.86–0.95]	<0.0001
ω-6 fatty acid (g)	1.23 [1.17–1.29]	<0.0001	0.98 [0.93–1.03]	0.3798
Cholesterol (mg)	1.57 [1.49–1.65]	<0.0001	1.32 [1.25–1.39]	<0.0001
Water (mL)	1.03 [0.99–1.09]	0.1784	0.76 [0.72–0.80]	<0.0001
Calcium (mg)	0.98 [0.93–1.03]	0.3384	0.82 [0.78–0.87]	<0.0001
Phosphate (mg)	1.08 [1.03–1.13]	0.0017	0.87 [0.82–0.91]	<0.0001
Sodium (mg)	1.41 [1.34–1.48]	<0.0001	1.18 [1.12–1.24]	<0.0001
Potassium (mg)	1.09 [1.04–1.14]	0.0005	0.88 [0.83–0.92]	<0.0001
Magnesium (mg)	1.05 [1.00–1.11]	0.0374	0.87 [0.82–0.91]	<0.0001
Iron (mg)	1.46 [1.39–1.53]	<0.0001	1.28 [1.22–1.35]	<0.0001
Zinc (mg)	1.50 [1.43–1.58]	<0.0001	1.26 [1.19–1.32]	<0.0001
Vitamin A (μg RAE)	1.05 [1.00–1.10]	0.0383	0.78 [0.74–0.82]	<0.0001
Carotene (μg)	1.03 [0.98–1.08]	0.2437	0.82 [0.78–0.86]	<0.0001
Vitamin C (mg)	0.90 [0.85–0.94]	<0.0001	0.73 [0.70–0.77]	<0.0001
Vitamin D (μg)	1.10 [1.05–1.15]	0.0001	1.01 [0.96–1.06]	0.7034
Vitamin E (mg α-TE)	1.14 [1.08–1.19]	<0.0001	0.86 [0.82–0.90]	<0.0001

^(1)^ Model 1: Non-Adjusted (Crude). ^(2)^ Model 2: Adjusted for age, BMI, residential area, educational level, household income, occupational category, near-work, current smoking, heavy alcohol drinking, menopause, medications, rheumatoid arthritis, osteoporosis, diabetes, thyroid disease, and refractive surgery. OR; odds ratio, CI; confidence interval.

## Data Availability

All data files are available from the Korea Centers for Disease Control and Prevention database through the following URL: https://knhanes.kdca.go.kr/knhanes/sub03/sub03_02_05.do (accessed on 3 November 2023). Individuals, including international researchers who have signed up for membership, can utilize raw data from this website. However, the data access process and user manual are written in Korean.

## Supplementary Materials

The following supporting information can be downloaded at: https://www.mdpi.com/article/10.3390/nu16030372/s1, Table S1: Average daily intake of dietary nutrients in subjects with and without dry eye syndrome.
