# Supplementary material for: Impact of Dietary Nutrients on the Prevalence of Dry Eye Syndrome among Korean Women Aged 40 and above: Evidence from the Korea National Health and Nutrition Examination Survey
Source: Nutrients. 2024 Jan 26;16(3):372. doi: 10.3390/nu16030372 (PMC10857437; doi:10.3390/nu16030372)
Supplement: Supplementary file 1 [file nutrients-16-00372-s001.zip › nutrients-2728710-supplementary.pdf]

Table S1. Average daily intake of dietary nutrients in subjects with and without dry eye syndrome

Nutrients	DES[1)] (n=7,134) Mean (SD)		Non-DES (n=85,754) Mean (SD)		p-value[2)]
Energy (kcal)	1699.78	580.68	1600.86	584.45	<.0001
Water (g)	1060.44	608.75	1002.68	529.17	<.0001
Carbohydrate (g)	263.45	97.42	259.71	99.77	0.0023
Protein (g)	64.71	28.84	58.43	25.77	<.0001
Fat (g)	41.98	23.86	35.14	23.33	<.0001
Saturated fatty acid (g)	12.46	8.19	10.41	7.81	<.0001
Monounsaturated fatty acid (g)	13.71	8.89	10.71	8.17	<.0001
Polyunsaturated fatty acid (g)	10.99	6.38	10.00	7.05	<.0001
N3 fatty acid (g)	1.70	1.31	1.85	1.78	<.0001
N6 fatty acid (g)	9.27	5.56	8.13	5.95	<.0001
Cholesterol (mg)	237.69	177.00	188.16	162.05	<.0001
Fiber (g)	28.01	13.17	27.89	13.69	0.4640
Sugar (g)	60.96	38.40	57.94	40.01	<.0001
Calcium (mg)	459.20	230.50	493.90	290.49	<.0001
Phosphate (mg)	1027.45	433.77	965.24	405.81	<.0001
Iron (mg)	9.62	4.30	8.91	4.94	<.0001
Sodium (mg)	3067.78	1551.92	2852.51	1565.19	<.0001
Potassium (mg)	2881.66	1460.99	2770.13	1348.32	<.0001
Vitamin A (μg RAE)	433.92	332.53	413.00	468.34	0.0002
Carotene (μg)	3807.54	3458.75	3443.23	3275.81	<.0001
Retinol (μg)	116.58	136.82	126.05	340.03	0.0195
Vitamin B1 (mg)	1.11	0.58	1.03	0.67	<.0001
Vitamin B2 (mg)	1.46	0.70	1.31	0.67	<.0001
Niacin (mg)	11.49	6.55	10.31	6.15	<.0001
Vitamin C (mg)	73.78	85.61	73.77	72.91	0.9916
Magnesium (mg)	321.21	139.55	307.97	133.67	<.0001
Zinc (mg)	10.09	4.08	9.22	4.00	<.0001
Vitamin D (μg)	2.63	2.81	2.92	6.53	0.0002
Vitamin E (mg α-TE)	6.83	3.40	6.38	4.13	<.0001
Folate (μg DFE)	336.01	187.71	331.70	166.04	0.0374

1) DES; Dry eye syndrome

2) Different between two groups at α=0.05 by ANCOVA test
